# Effectiveness of V-Go^®^ for Patients with Type 2 Diabetes in a Real-World Setting: A Prospective Observational Study

**DOI:** 10.1007/s40801-019-00173-8

**Published:** 2019-12-12

**Authors:** George Grunberger, Cheryl R. Rosenfeld, Bruce W. Bode, Scott D. Abbott, Carla Nikkel, Leon Shi, Poul Strange

**Affiliations:** 1 Grunberger Diabetes Institute, Bloomfield Hills, MI USA; 2 Internal Medicine and Molecular Medicine and Genetics, Wayne State University School of Medicine, Oakland University William Beaumont School of Medicine, Detroit, USA; 3 North Jersey Endocrine Consultants, LLC, Parsippany, NJ USA; 4 Touro College of Osteopathic Medicine, New York, USA; 5 Atlanta Diabetes Associates, Atlanta, GA USA; 6 Valeritas, Inc., Bridgewater, NJ USA; 7 Integrated Medical Development, Princeton Junction, NJ USA

## Abstract

**Background:**

V-Go is a wearable, patch-like, 24-h insulin delivery device that delivers both a continuous preset basal rate and on-demand bolus dosing. The aim of this study was to observe glycemic control, insulin dosing, and hypoglycemia risk in patients switched to V-Go in a real-world setting. The primary objective was to compare change in mean hemoglobin A1c (HbA1c) from baseline to the end of V-Go use.

**Methods:**

This prospective, open-label, multicenter study recruited patients with type 2 diabetes (T2D) and suboptimal glycemic control (HbA1c ≥ 7%) across 28 centers. Efficacy analyses were conducted for all patients with a post-baseline HbA1c and results stratified based on prior antihyperglycemic medication therapies. Insulin dosing was at the discretion of the health care provider and the protocol did not mandate glycemic targets. Treatment satisfaction surveys were utilized to gain patient feedback on the use of V-Go.

**Results:**

One hundred eighty-eight patients were enrolled in the study, among whom 140 patients had a valid post-baseline HbA1c and were included in the primary efficacy analysis. Use of V-Go resulted in a change of − 0.64%; (*P* = 0.003) in HbA1c from baseline, and in those prescribed insulin, the total daily dose of insulin was decreased by 12 units/day (*P* < 0.0001). Twenty-two patients (12%) reported hypoglycemic events (≤ 70 mg/dL), with an event rate of 1.51 events/patient/year.

**Conclusion:**

In a T2D population with suboptimal HbA1c, initiating V-Go therapy in a real-world setting significantly improved glycemic control and led to significant insulin dose reductions.

ClinicalTrial.gov registry identifier: NCT01326598.

## Key Points

**Table:** 

Initiating or managing intensified insulin therapy can be challenging due to the complexity of managing multiple injections, interference with daily living, and injection discomfort.
Wearable patch-like insulin delivery options can reduce the burdens associated with intensified insulin therapy and are nationally recognized as an alternative to insulin pens or syringes for insulin administration; however, prospective data are limited and are needed to evaluate the effectiveness and safety of patch-like insulin delivery methods.
In this study, the V-Go wearable insulin delivery device demonstrated effectiveness and was well tolerated in patients with T2D, resulting in high treatment satisfaction compared to previous diabetes therapies.

## Introduction

Patients with T2D are at risk for complications with increasing HbA1c, making glycemic control a priority. When glycemic targets are not achieved with lifestyle changes and oral agents, intensification of therapy to include injectable therapy is recommended. Insulin is the most potent antihyperglycemic therapy, and adding basal insulin is the preferred method when insulin therapy is initiated [1]. The implementation of a rigorously followed basal insulin titration algorithm in the management of T2D has demonstrated good results in improving HbA1c [2], although higher doses of basal insulin have been shown to have diminishing returns. Pooled data from 15 randomized treat-to-target trials in patients treated with basal insulin found that titrating basal doses above 0.5 units/kg did not result in further HbA1c or fasting plasma glucose improvement and increased the risk of hypoglycemia [3]. Guidelines support the consideration of prandial insulin boluses when basal insulin therapy fails to provide glucose control and the total daily dose (TDD) of basal insulin is greater than 0.5 units/kg [1, 4].

Basal-bolus regimens have proven effective, but insulin intensification is often delayed. Managing multiple injections can be disruptive to daily activities and it can be difficult to administer insulin injections discreetly. The unfortunate social stigma or ‘fear of embarrassment in public’ was found to be one of the most important reasons that people are not receptive to insulin therapy [5]. Despite the advances in insulin delivery technology, including insulin pen devices and electronic insulin infusion pumps, administering mealtime insulin still requires either extra injections or, for pumps, the need to pay extra attention to the technology involved. Simplification of basal-bolus insulin delivery is warranted to increase adoption of this effective treatment regimen by patients and physicians. Wearable patch-like devices can simplify basal-bolus insulin therapy in patients with T2D by reducing the number of required injections and the complexity of managing multiple insulins. These devices have recently been recognized by the American Diabetes Association as a delivery alternative to insulin pens and syringes [6].

The V-Go wearable insulin delivery device is the first fully disposable, 24-h wearable patch-like device for the delivery of basal-bolus insulin therapy in adult patients requiring insulin. Once filled and applied to the skin, the device delivers continuous subcutaneous U-100 fast-acting insulin (insulin lispro, rDNA origin or insulin aspart, rDNA origin) in preset basal rates of 20, 30, or 40 units (U) in one 24-h period, as well as on-demand bolus dosing of up to 36 U in 2 U increments. V-Go adheres to the skin using a hypoallergenic non-latex adhesive. After 24 h, the patient presses the needle release button, which retracts the needle back into the V-Go, allowing the removal and disposal of the device used before a new V-Go is applied and initiated [7]. Patients have reported the V-Go device to be discreet and easy to use, and the basal and bolus capability of the device eliminates the need for multiple injections, all of which may help improve patient compliance with insulin therapy [8, 9]. Evidence has supported an improvement in glycemic control with V-Go [9–17]; however, prospective data are lacking, and further evidence is warranted to understand the impact of V-Go across a wide range of patients.

## Methods

### Study Design

This trial was a prospective, observational, open-label, multicenter study of patients with a diagnosis of T2D and HbA1c ≥ 7% (53 mmol/mol) who were switched to V-Go in 28 private practice endocrinology and internal medicine clinics across the United States. Recruitment and enrollment of patients from the clinics’ patient populations occurred over a 14-month period. The study was initiated prior to the commercial launch of V-Go with the aim of observing the effect in a real-world clinical setting of instituting V-Go therapy compared to prior treatment regimens on glycemic control, insulin dose requirements, hypoglycemia risk, weight change, and other possible adverse effects during circumstances as close to normal clinical practice as possible.

Patients were identified and recruited by participating sites if the patient met eligibility criteria and was willing to try a new insulin delivery device. After screening, enrolled patients were instructed to continue current antihyperglycemic medications and normal activities for a 4- to 6-week run-in period before V-Go was initiated. This design accounted for possible improvements in HbA1c due to clinical study effects unrelated to V-Go. Following the run-in period, baseline (month 0) HbA1c values were captured and patients were trained on how to fill, wear and appropriately use V-Go for insulin administration. Patients also received education in dosing insulin before the start of each meal, and for patients who had not used rapid acting insulin before, the immediate risk of hypoglycemia was stressed. Diet and exercise reinforcement by the investigator or other qualified person was reviewed with patients. Education pertaining to lifestyle management beyond the baseline visit was not stipulated in the protocol. Patients returned for physician visits and data collection at months 3, 6, 9 and 12 as long as they continued in the study, irrespective of whether they were treated with V-Go during the full period. Patient satisfaction surveys were conducted after 1 month of V-Go initiation by phone in addition to each physician office visit. Data were recorded by the clinics and entered in a web-based electronic data capture system by the clinic staff. Sponsor monitors visited the clinics periodically to check study conduct and data. Consolidated data were subsequently reviewed to identify possible outliers for further checks before database lock. The study was observational in nature, so there were no protocol-mandated instructions to guide therapy, and forced insulin titration measures were not implemented. Aside from using V-Go within the cleared label, no limitations were imposed on concomitant therapy. Protocol procedures allowed for a 3-month supply of V-Go devices by the manufacturer, which was amended to 6 months after the study was in progress to accommodate limited accessibility and coverage of V-Go by insurance providers due to the introduction of V-Go as a new product.

### Patients

The inclusion and exclusion criteria were minimally restrictive given the real-world aspect to this study. Inclusion criteria to qualify for participation included: age between 21 and 80 years old; diagnosed with T2D at least 12 months prior; on an antihyperglycemic therapy regimen for at least a month; willing to self-monitor blood glucose at least twice a day; and HbA1c greater than or equal to 7.0% (53 mmol/mol) at screening. Exclusion criteria included: acute infection with fever; serum creatinine > 265.2 μmol/L (3.0 mg/dL) if not on metformin, or if on metformin, serum creatinine > 123.8 μmol/L (1.4 mg/dL) (females) and > 132.6 μmol/L (1.5 mg/dL) (males) within the last 6 months; pregnancy or the intention to become pregnant; any medical history of malignant melanoma or breast cancer; medical history of any cancers within the last 5 years (except adequately treated basal cell carcinoma or cervical carcinoma in situ); history of alcohol or drug abuse within the last year; participation in other clinical trials involving receipt of the investigational drug within the last 30 days; unwillingness or inability to comply with study procedures; requiring regular adjustments to the basal rate during a 24-h period; requiring insulin adjustments at meals of less-than 2-unit increments; or history of hypersensitivity to adhesives.

The population included patients stratified for analysis based on their prior antihyperglycemic therapy as follows: stratum 1 (S1), oral antihyperglycemic drugs (OADs) only; stratum 2 (S2), OADs + non-insulin injectable; stratum 3 (S3), once/twice-daily long-acting insulin injection ± OADs/non-insulin injectable; stratum 4 (S4), 1–3 daily premix insulin injections ± OADs/non-insulin injectable; and stratum 5 (S5), multiple daily injection (MDI) therapy with 3+ daily insulin injections ± OADs/non-insulin injectable.

### Effectiveness Assessments

The primary objective was to compare the mean change in glycemic control measured by HbA1c from baseline to the end of V-Go use, and was conducted in all patients who had at least one post-baseline HbA1c on V-Go. Secondary objectives for this study included determining change in HbA1c and insulin dose from baseline at particular time points. The analyses presented here focus on 3- and 6-month data to account for limitations imposed by cost and availability constraints of the device at the time of the study. Subanalyses for efficacy based on prior antihyperglycemic therapy are focused on strata 3–5, which included a majority of the trial patients and the most natural population for this device. The HbA1c and insulin dose requirements of patients who continued treatment with V-Go compared to those who switched to other therapies but continued in the study as the comparison group were also summarized.

### Safety Assessments

Safety was evaluated through analyses of hypoglycemic events and adverse events. Hypoglycemia was reported as both incidence and prevalence. Hypoglycemia was analyzed based on the following definitions: severe—an event requiring assistance to administer carbohydrate, glucagon, or other resuscitative actions, and a confirmation from either plasma glucose ≤ 36 mg/dL (2 mmol/L) or prompt neurological recovery upon treatment to restore glucose to normal; documented—symptoms of hypoglycemia confirmed with a measured plasma glucose ≤ 70 mg/dL (3.9 mmol/L).

### Patient Assessments: Treatment Satisfaction

Patients using V-Go were asked to complete treatment satisfaction surveys by phone 1 month after initiating V-Go and at the start of each physician visit. Questions were answered by assigning values on a scale of 1–10 (with 1 being the worst and 10 being the best) as follows: (a) How comfortable was the V-Go? (b) How discreet was the V-Go to wear? (c) How do you feel physically? (d) How do you feel mentally? (e) How does the V-Go compare to previous therapies? Patient responses were analyzed descriptively as no baseline data were available for comparative purposes.

### Statistical Analyses

All effectiveness and safety analyses were performed on the safety population consisting of all patients who were initiated on V-Go at month 0 and utilized at least one V-Go and had at least one post-product safety assessment. The primary analysis used an ANOVA model involving all patients from the safety population with at least one post-baseline HbA1c measurement, and used the least-squares mean change of HbA1c from baseline at month 0 to the last time point of V-Go use as the endpoint. The mixed model included fixed effects: strata, study site, visit, and the corresponding interactions, and the best fit within patient covariance structure was based on the Akaike criterion. A similar analysis was applied when evaluating the change from baseline based on prior antihyperglycemic therapy strata, except that therapy strata was excluded as an effect. Sites with low enrollment were combined. To compare change from baseline between patients continuing and discontinuing V-Go use, a mixed model with V-Go status and strata as fixed effects and he corresponding interaction as a covariate was employed. Safety was evaluated by analyzing hypoglycemia and adverse events. The sample size was initially planned as 33 per stratum, yielding 80% power to detect a change from baseline HbA1c of 0.5% units given a standard deviation of 1.0% units. Study enrollment differed widely across the strata, so rather than stopping enrollment in the faster-recruiting strata, the protocol was amended to allow up to 270 subjects in total, and was eventually stopped at 233. Statistical analyses were performed using SAS 9.1 or higher.

## Results

### Patient Disposition and Baseline Characteristics

Two hundred thirty-three patients were screened, among whom 188 patients with T2D were enrolled in the study, with 187 patients completing the run-in period and 140 patients (74%) having at least one valid post-baseline HbA1c on V-Go for inclusion in the primary analysis. Medical history conditions reported by > 10% were hypertension (59%), hyperlipidemia (56%), coronary artery disease (26%), neuropathy (21%), arthritis (18%), gastroesophageal reflux disease (13%), and depression (12%). The patient dispositions at the 3- and 6-month time points reported here were as follows: by 3 months, 153 patients (81%) continued in the study, with 141 patients on V-Go; by 6 months, 133 patients (71%) remained in the study, with 111 patients on V-Go. Twelve patients were using therapies other than V-Go for glucose control at 3 months, and 22 patients were doing so at 6 months. By the end of the study, 112 patients (60%) remained in the study, among whom 66 patients were on V-Go and 46 patients were using therapies other than V-Go to treat their diabetes. Major reasons for discontinuation included cost (18.1%) and adverse effects (17.6%), which were mainly hyperglycemia or skin reactions. Within the population who discontinued due to cost, the highest rates of dropout were seen between 3 and 9 months, corresponding to the period when V-Go devices were no longer provided. Less than 7% of the population reported V-Go discontinuation due to user problems such as inconvenience, lack of comfort, or not liking the product.

Within the overall population, patients were stratified by their prior antihyperglycemic therapy regimens. Sample sizes were relatively small for strata 1–2, so subanalyses focused on strata 3–5: those patients using insulin prior to the study, who comprised 90% of the study population and are a natural population for this device. Within this cohort, patients on MDI (S5) made up 42% of the total population.

Demographic and baseline characteristics of the patients enrolled in the study are presented in Tables 1 and 2. The age of participants ranged from 26 to 70 years. The patient population enrolled in the study can be categorized as advanced patients with diabetes based on the medical conditions present and years since diagnosis. Mean HbA1c at the screening visit and at the end of the 4- to 6-week run-in period (baseline: month 0) is shown in Table 2. There was a mean overall − 0.15% reduction in HbA1c from screening to the end of the run-in period. Across all patients, the mean basal dose ranged from 0 to 150 U/day and the TDD ranged from 0 to 238 U/day at baseline. In the MDI (S5) population ,the TDD ranged from 25 to 238 U/day.

**Table 1:** Baseline patient demographics for all patients stratified according to prior insulin therapy

	All patients *N* = 188	Stratum 3 *n* = 59	Stratum 4 *n* = 31	Stratum 5 *n* = 79
Age (years)	59.1 ± 10.4	58.2 ± 10.1	60.9 ± 11.9	59.7 ± 10.4
Gender [female/male (%)]	47/53	39/61	65/35	48/52
Race				
Asian	1 (< 1)	0 (0)	0 (0)	1 (1)
Black/African	17 (9)	4 (7)	4 (13)	8 (10)
Other	5 (3)	4 (7)	0 (0)	1 (1)
White/Caucasian	165 (88)	51 (86)	27 (87)	69 (87)
Hispanic ethnicity	15 (8)	6 (10)	5 (16)	2 (3)
DM duration (years)	14 ± 8.0	14 ± 7.9	15 ± 8.6	14 ± 8.2
Insurance				
Cash	8 (4)	4 (7)	1 (3)	2 (3)
Commercial	106 (56)	33 (56)	13 (42)	51 (65)
Medicaid	4 (2)	0 (0)	2 (7)	1 (1)
Medicare	67 (36)	20 (34)	14 (45)	25 (32)
Other	3 (2)	2 (3)	1 (3)	0 (0)

Stratum 3 (S3) = once/twice-daily long-acting insulin injection ± OADs/non-insulin injectable; stratum 4 (S4) = 1–3 daily premix insulin injections ± OADs/non-insulin injectable; and stratum 5 (S5) = multiple daily injection (MDI) therapy with 3+ daily insulin injections ± OADs/non-insulin injectable. ‘All patients (*N* = 188)’ includes stratum 1 (S1) = OADs only; stratum 2 (S2) = OADs + non-insulin injectable

Data are mean ± SD or *n* (%) unless otherwise noted

*DM* diabetes mellitus, *OADs* oral antihyperglycemic drugs, *SD* standard deviation

**Table 2:** Baseline clinical characteristics for all patients stratified according to prior insulin therapy

	All patients *N* = 188	Stratum 3 *n* = 59	Stratum 4 *n* = 31	Stratum 5 *n* = 79
Weight (kg)	103.9 ± 21.8	105.7 ± 22.2	96.6 ± 21.8	104.8 ± 22.2
HbA1c (%)				
Screening	8.88 ± 1.58	9.05 ± 1.54	9.42 ± 2.03	8.68 ± 1.50
Baseline (month 0)^a^	8.73 ± 1.54	8.71 ± 1.40	9.33 ± 1.84	8.65 ± 1.59
Insulin (U/day)				
Basal	44 ± 30	50 ± 24	53 ± 25	47 ± 31
Bolus	22 ± 27	6 ± 20	22 ± 10	39 ± 29
Total daily dose	66 ± 46	56 ± 31	75 ± 35	87 ± 49
Antihyperglycemic medications^b^				
Non-insulin injectables	38 (20)	11 (19)	4 (13)	17 (22)
Oral antihyperglycemic drugs	126 (67)	43 (73)	22 (71)	42 (53)

Stratum 3 (S3) = once/twice-daily long-acting insulin injection ± OADs/non-insulin injectable; stratum 4 (S4) = 1–3 daily premix insulin injections ± OADs/ non-insulin injectable; and stratum 5 (S5) = multiple daily injection (MDI) therapy with 3+ daily insulin injections ± OADs/non-insulin injectable. ‘All patients (*N* = 188)’ includes stratum 1 (S1) =  OADs only; stratum 2 (S2) = OADs + non-insulin injectable

Data are mean ± SD or *n* (%)

*OADs* oral antihyperglycemic drugs, *SD* standard deviation

^a^Baseline HbA1c was not available for 1 patient in stratum 4

^b^Summarized as the number and percent of patients prescribed ≥ 1 non-insulin injectable and/or ≥ oral antihyperglycemic drug

### Effectiveness

In the primary efficacy analysis, glycemic control, measured as the change in HbA1c, improved significantly when evaluating the change from month 0 to the last time point of V-Go use in patients (*N* = 140) with at least one post-baseline HbA1c measurement. The average duration of V-Go use was 278 days, with a mean change in HbA1c of − 0.64% (95% CI − 0.99, − 0.30; *P* = 0.003).

**Fig. 1:**
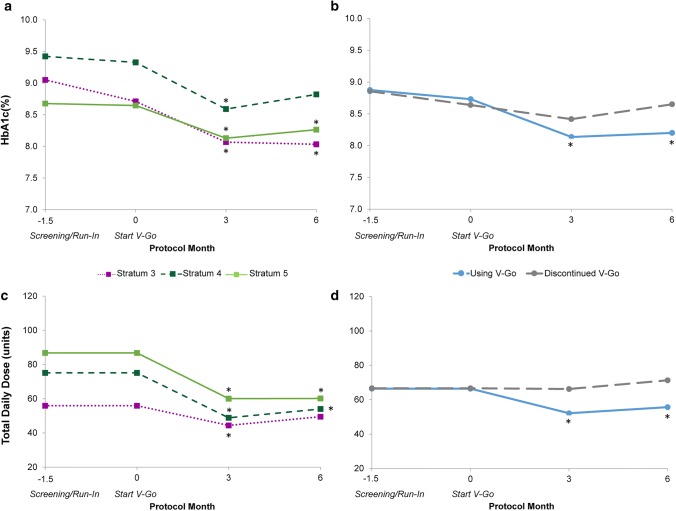
Comparison of change in HbA1c over time by strata and V-Go use. **a** Mean HbA1c from screening to month 6; **b** HbA1c for all patients using V-Go vs. patients discontinuing V-Go; **c** mean total daily dose from screening to month 6; **d** total daily dose of all patients using V-Go vs. patients discontinuing V-Go. Stratum 3 (S3) = once/twice-daily long-acting insulin injection ± OADs/non-insulin injectable; stratum 4 (S4) = 1–3 daily premix insulin injections ± OADs/ non-insulin injectable; and stratum 5 (S5) = multiple daily injection (MDI) therapy with 3+ daily insulin injections ± OADs/non-insulin injectable. *OADs* oral antihyperglycemic drugs

Secondary objectives included assessing changes for particular time points, and were focused on 3- and 6-month changes. Changes in HbA1c from baseline based on continuation of V-Go use at each time point are reported in Table 3 with stratification for S3, S4, and S5. After 6 months of V-Go use, patients in S3 (long-acting insulin users) showed the greatest decrease from baseline in HbA1c of − 0.59% (95% CI − 0.91, − 0.26; *P* = 0.0005). The decreases in HbA1c from baseline were accompanied by a significant drop in TDD at both 3 and 6 months for S4 and S5 and at 3 months for S3, as illustrated in Fig. 1a, c. The largest decrease in TDD was seen in the MDI (S5) population, with a mean decrease in TDD of 26.6 U/day (86.8–60.2 U/day) from screening to 6 months.

**Table 3:** Patients continuing V-Go at 3 and 6 months stratified according to prior insulin therapy

	Months	*n*	HbA1c change	95% CI	*P* value
Stratum 3	3	46	− 0.65 ± 0.14	− 0.93, − 0.37	< 0.0001
	6	34	− 0.59 ± 0.16	− 0.91, − 0.26	0.0005
Stratum 4	3	21	− 0.63 ± 0.24	− 1.11, − 0.15	0.0114
	6	18	− 0.48 ± 0.26	− 1.00, 0.04	0.0696
Stratum 5	3	58	− 0.62 ± 0.15	− 0.92, − 0.31	< 0.0001
	6	47	− 0.45 ± 0.17	− 0.79, − 0.11	0.0098
All strata (1–5)	3	138	− 0.65 ± 0.09	− 0.83, − 0.47	< 0.0001
	6	110	− 0.55 ± 0.10	− 0.75, − 0.35	< 0.0001

Stratum 3 (S3) = once/twice-daily long-acting insulin injection ± OADs/non-insulin injectable; stratum 4 (S4) = 1–3 daily premix insulin injections ± OADs/ non-insulin injectable; and stratum 5 (S5) = multiple daily injection (MDI) therapy with 3+ daily insulin injections ± OADs/non-insulin injectable

Data are mean ± SE

*CI* confidence interval, *OAD* oral antihyperglycemic drugs

**Fig. 2:**
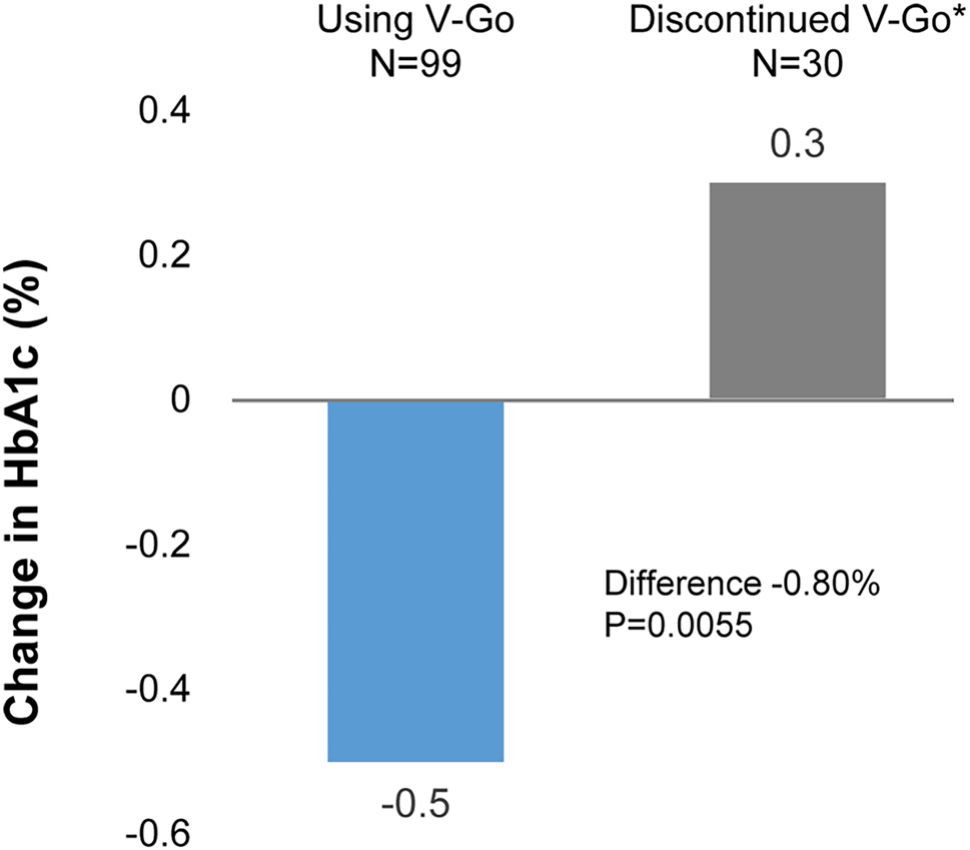
Comparison of change in HbA1c from baseline in patients from strata 3, 4, 5 who were using V-Go at month 6 versus patients who discontinued V-Go and resumed other antihyperglycemic therapies for 6 months. Stratum 3 (S3) = once/twice-daily long-acting insulin injection ± OADs/non-insulin injectable; stratum 4 (S4) = 1–3 daily premix insulin injections ± OADs/non-insulin injectable; and stratum 5 (S5) = multiple daily injection (MDI) therapy with 3+ daily insulin injections ± OADs/non-insulin injectable. *For patients who discontinued V-Go, month numbers were grouped into 3-month intervals for the purposes of summarization. *OADs* oral antihyperglycemic drugs

Patients who discontinued V-Go use were encouraged to continue with the follow-up in the study and served as a natural comparison group for patients who remained on V-Go. Comparisons of all patients (S1, S2, S3, S4, S5) who continued V-Go with those who discontinued V-Go are shown in Fig. 1b and d. A significant reduction in HbA1c was observed at 6 months compared to baseline, with a concurrent reduction in the TDD of insulin from 66.4 to 55.7 U/day in patients with continued V-Go use. This finding differed substantially from those who discontinued V-Go, where averages for both HbA1c and TDD were similar or higher at 6 months compared to baseline values. A separate analysis was performed to compare the change from baseline HbA1c in patients from strata 3–5 with 6 months of continued V-Go use (*n* = 99) to patients with 6 months of resumed use after switching to other antihyperglycemic therapies (*n* = 30) to evaluate the change in HbA1c based on a similar persistence with therapy. Results from this analysis are shown in Fig. 2 and demonstrate that continued V-Go use resulted in better glycemic control compared to those who discontinued V-Go to resume other therapies.

### Patient Treatment Satisfaction

**Fig. 3:**
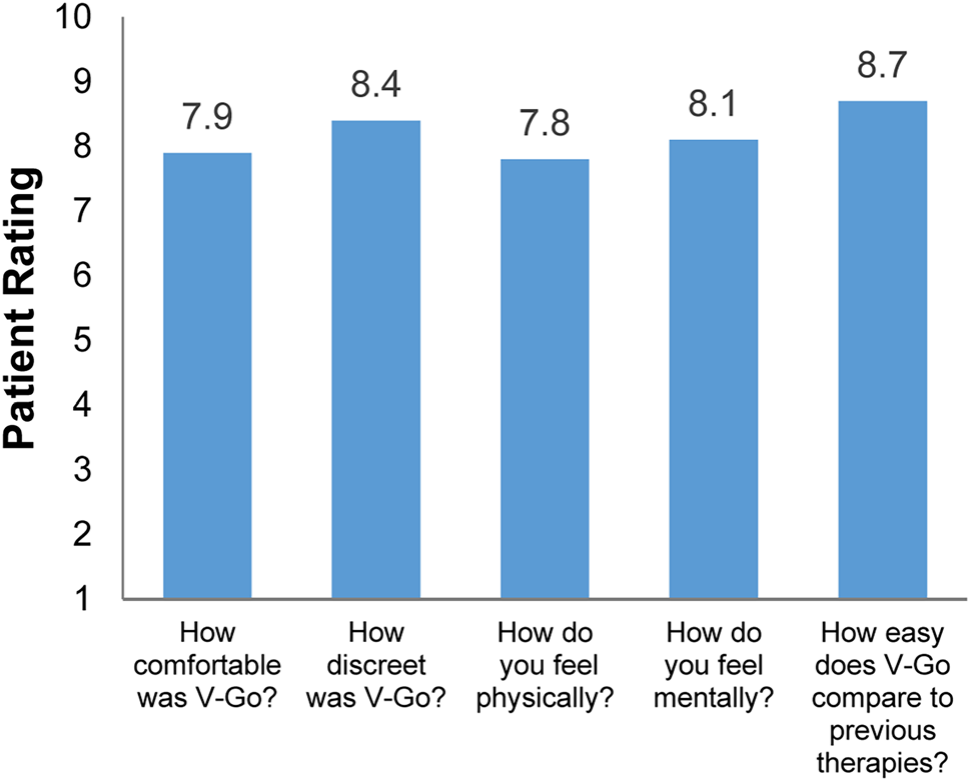
Patient treatment satisfaction ratings for V-Go. Patients (*N* = 171) answered questions by assigning a value on a scale of 1–10 (with 1 being the worst and 10 being the best) from month 1 of the study to the end of the study. All patient surveys are included, regardless of the completion of the study or the continued use of V-Go

One hundred seventy-one patients between month 1 and the end of the study responded to questionnaires designed to gain patient perspectives on the ease, comfort, and health-related feelings associated with V-Go use. All patient surveys are included regardless of the completion of the study or the continued use of V-Go. The ratings were generally high in regards to treatment satisfaction with the use of V-Go, and are summarized in Fig. 3. Patient impressions were similar to those for the overall population when stratified.

### Safety

Twenty-two patients (12%) reported a hypoglycemic event (≤ 70 mg/dL) while on V-Go, including one severe event at the end of 12 months. All events resolved without sequelae. The rate of hypoglycemic events was low at 1.51 events per patient per year. One patient on V-Go reported a serious adverse event (SAE), hyperglycemia, that was deemed to be possibly related to V-Go. Three patients reported V-Go-related severe adverse events: two patients with skin rashes and one with hyperglycemia. Both the SAE and the severe adverse events resolved without sequelae.

Thirty-three patients (17.5%) discontinued V-Go use due to V-Go-related adverse effects. Within this group, two patients (1.1%) discontinued due to hypoglycemia; both patients had been prescribed MDI at baseline. One patient (0.5%) discontinued due to a skin infection, 11 patients (5.9%) due to skin reactions (such as redness), and 19 patients (10.1%) due to reported hyperglycemia. Discontinuations for adverse events were distributed evenly across the evaluation period.

Changes in weight were assessed for the duration of the study. Mean values of baseline weight are reported in Table 2. For the patients using V-Go, the change in body weight was + 1.04 kg (*P* = 0.0504) and + 1.63 kg (*P* = 0.0213) at 6 and 12 months, respectively. In patients discontinuing V-Go, the change in body weight was + 0.35 kg (*P* = NS) and 0.96 kg (*P* = NS) at 6 and 12 months, respectively.

## Discussion

Approximately 8 million people with diabetes are prescribed insulin in the United States, highlighting the importance of the devices that deliver insulin [18]. The viewpoint of insulin therapy differs greatly between T2D and type 1 diabetes (T1D). In T1D diabetes, exogenous insulin therapy is required to sustain life; in T2M, insulin may be viewed by patients as optional to delay complications. These differences in mindset may impact the acceptance of and compliance with insulin therapy. The majority of people with diabetes requiring basal-bolus therapy depend on an insulin pen or syringe to deliver multiple injections of insulin per day, which can be burdensome. Electronic insulin infusion pumps can also be used, but are typically recommended in people with T1D due to advanced functions that may benefit this population, including variable basal insulin rates, administration of very small doses for people who are sensitive to insulin, and dosing calculators to determine required doses based on carbohydrate counting. In people with T2D, these advanced pump features are typically not warranted, and the complexity of an electronic insulin infusion pump and the high associated upfront costs can be a barrier to use. Simplicity of therapy can facilitate adoption of and persistence with therapy in a T2D population. Disposable wearable basal-bolus insulin delivery devices such as V-Go offer the benefits of continuous infusion, similar to a pump, a comparable price point to insulin pen therapy, and the removal of the burden of multiple injections. These patch-like devices are now included in the American Diabetes Association’s current clinical practice recommendations as an alternative to insulin pens and syringes, but prospective data evaluating the use of these devices in a real-world practice setting are limited. To our knowledge, only two prospective studies [16, 17] have been published, and both are based on short-term use. This observational study, designed to mimic real-world conditions as closely as possible, explored the effectiveness and safety of V-Go use in patients with suboptimally controlled T2D. Patients enrolled in the study were on a variety of antihyperglycemic regimens prior to the start of the study. While this trial included patients previously not on insulin (S1, S2), the most “natural” population for this device comprises those patients requiring insulin prior to V-Go initiation (S3, S4, and S5).

V-Go effectiveness was observed in this study, with a statistically significant reduction in HbA1c during V-Go use that was accompanied by lower insulin requirements in patients previously prescribed insulin. An overall mean change of − 0.64% in HbA1c from baseline was observed in patients who initiated V-Go. These findings are clinically relevant considering the advanced stage of diabetes in this population and the fact that over 40% of the patient population was intensively treated with insulin and prescribed a basal-bolus regimen at baseline. With disease progression and disruption of physiologic processes, the treatment of patients with advanced diabetes becomes more challenging. Improved dosing and titration recommendations toward prespecified glycemic targets would have likely resulted in even more incremental improvements in glycemic control.

Among patients who were prescribed insulin prior to the start of the study, the between-group difference in change in HbA1c after 6 months was − 0.80% in favor of those patients continuing V-Go versus those who discontinued V-Go. It is interesting to note that patients who discontinued V-Go had slightly lower baseline HbA1c values and similar TDD requirements compared to those who continued on V-Go, suggesting that baseline characteristics did not contribute to their lack of clinical benefit. Findings from this trial are comparable to previously reported evidence. A retrospective study that employed a similar comparison of patients who persisted with V-Go versus those who discontinued V-Go to resume conventional insulin therapy demonstrated a significant between-group difference (1.2%; *P* = 0.003) favoring V-Go when evaluating change in HbA1c from baseline after 5 months of persistence with therapy [14]. Cziraky and colleagues reported findings from a randomized controlled trial comparing V-Go to standard treatment optimization (STO). In this study, both therapies significantly reduced HbA1c from baseline V-Go (− 1.0%, *P* < 0.001) and STO (− 0.5%, *P* < 0.001); however, V-Go resulted in a significantly (*P* = 0.002) larger reduction [16].

Patient impressions of V-Go were generally high across all questions surveyed in this study, which is consistent with previous treatment satisfaction surveys [9, 16]. V-Go ease of use compared to previous therapy received the highest rating (8.7 out of 10), which is significant considering that regimen complexity and patient satisfaction can both impact patient compliance, which is critical to improving glycemic control [19].

Overall, V-Go was well accepted and tolerated in this study. Only one patient experienced a serious adverse event (SAE) related to V-Go use, and 12% of the population reported a hypoglycemic event during the trial. Only one of the hypoglycemic events was documented as severe, and all events resolved with no sequelae.

Although this research achieved its aims, there are limitations to this analysis. First and foremost, observational study designs only allow for an association of the given condition with the outcome, and may introduce bias in regards to patient selection when eligibility criteria are minimally restrictive, as in this study. Although observational studies offer insight into the effectiveness of a therapy in routine clinical practice, a randomized controlled trial is the optimal design for drawing inferences about causation. Lack of insurance coverage for V-Go was found to play a key role in this trial and resulted in high attrition rates. This trial was initiated approximately 5–8 months prior to the commercial launch of V-Go, at which time there were no insurance reimbursement programs in place. This lack of insurance coverage and access to V-Go impacted the majority of the enrolled patients with regard to persistence with V-Go. When patients no longer received V-Go devices from the sponsor, high dropout rates were seen. A subanalysis evaluated the change in HbA1c in patients who stayed in the study but had discontinued V-Go due to cost. This analysis showed a 1.48% mean increase in HbA1c from the time of discontinuation to 6 months postdiscontinuation (*n* = 6). However, due to the small number of patients who remained in the study after discontinuation and the potential bias introduced because V-Go devices were not provided after 6 months, this result cannot be generalized to the full population of those who discontinued. Second, to be included in the primary analysis, patients were required to have at least one HbA1c measurement after V-Go initiation, so the data may not be representative of all patients initiating V-Go therapy. Finally, the reported prevalence and incidence of hypoglycemia may have differed, as patient self-reporting was used in the findings.

Strengths of our study include a longer study duration and a patient population who were prescribed a broad spectrum of antihyperglycemic medications at baseline, both of which are needed to address important gaps in the current literature. Additional robust clinical evidence, including randomized controlled studies, are planned to better understand the benefits of V-Go and to support the use of this technology.

## Conclusion

This study assessed the clinical impact of V-Go for insulin delivery in a real-world clinical setting without the influence of mandated glycemic targets or forced insulin titration algorithms. Use of V-Go resulted in significantly improved glycemic control across the patient population, and did so with significantly less insulin among most patients with prior insulin use. Satisfaction with V-Go was high, and the device was well tolerated and had an acceptable safety profile.

## Data Availability

The datasets generated during and/or analyzed during the current study are available from the corresponding author on reasonable request.
